# Supplementary material for: Does the 11-year solar cycle affect lake and river ice phenology?
Source: PLoS One. 2023 Dec 13;18(12):e0294995. doi: 10.1371/journal.pone.0294995 (PMC10718462; doi:10.1371/journal.pone.0294995)
Supplement: S1 Table — (DOCX) [file pone.0294995.s001.docx]

**Does the 11-Year Solar Cycle Affect Lake and River Ice Phenology? (Supporting Information)**

|  | **Ice-On** | **Ice-Off** |
| --- | --- | --- |
| **20+ years** | 371 | 468 |
| **40+ years** | 185 | 290 |
| **60+ years** | 112 | 205 |
| **80+ years** | 60 | 140 |
| **100+ years** | 39 | 79 |

**Table S1:** The number of locations with at least 20 years of data, at least 40 years of data, etc.
