# Supplementary material for: Does the 11-year solar cycle affect lake and river ice phenology?
Source: PLoS One. 2023 Dec 13;18(12):e0294995. doi: 10.1371/journal.pone.0294995 (PMC10718462; doi:10.1371/journal.pone.0294995)
Supplement: S2 Table — (DOCX) [file pone.0294995.s002.docx]

**Does the 11-Year Solar Cycle Affect Lake and River Ice Phenology? (Supporting Information)**

| **Decade** | **Locations with Data** | **Decade** | **Locations with Data** |
| --- | --- | --- | --- |
| 1820s | 6 | 1920s | 133 |
| 1830s | 8 | 1930s | 173 |
| 1840s | 9 | 1940s | 221 |
| 1850s | 15 | 1950s | 386 |
| 1860s | 25 | 1960s | 484 |
| 1870s | 30 | 1970s | 541 |
| 1880s | 40 | 1980s | 552 |
| 1890s | 51 | 1990s | 327 |
| 1900s | 92 | 2000s | 158 |
| 1910s | 123 | 2010s | 102 |

**Table S2**. The number of locations with ice-on data available in a given decade.
