# Supplementary material for: Does the 11-year solar cycle affect lake and river ice phenology?
Source: PLoS One. 2023 Dec 13;18(12):e0294995. doi: 10.1371/journal.pone.0294995 (PMC10718462; doi:10.1371/journal.pone.0294995)
Supplement: S3 Table — (DOCX) [file pone.0294995.s003.docx]

**Does the 11-Year Solar Cycle Affect Lake and River Ice Phenology? (Supporting Information)**

| **Decade** | **Locations with Data** | **Decade** | **Locations with Data** |
| --- | --- | --- | --- |
| 1820s | 8 | 1920s | 193 |
| 1830s | 14 | 1930s | 239 |
| 1840s | 18 | 1940s | 304 |
| 1850s | 24 | 1950s | 498 |
| 1860s | 33 | 1960s | 608 |
| 1870s | 46 | 1970s | 635 |
| 1880s | 63 | 1980s | 660 |
| 1890s | 76 | 1990s | 446 |
| 1900s | 102 | 2000s | 230 |
| 1910s | 166 | 2010s | 141 |

**Table S3**. The number of locations with ice-off data available in a given decade.
